# Comparison of two data collection processes in clinical studies: electronic and paper case report forms

**DOI:** 10.1186/1471-2288-14-7

**Published:** 2014-01-17

**Authors:** Anaïs Le Jeannic, Céline Quelen, Corinne Alberti, Isabelle Durand-Zaleski

**Affiliations:** 1 Département de la recherche clinique et du développement, AP-HP, Groupe hospitalier Cochin Hôtel-Dieu, URC Économie de la Santé Ile de France, F-75004 Paris, France; 2 AP-HP, Hôpital Robert Debré, Unité d’Épidémiologie clinique, Groupe Hospitalier Robert Debré, 48, Bld Sérurier, F-75019 Paris, France; 3 Université Paris Diderot, PRES Sorbonne Paris Cité, F-75019 Paris, France; 4 Service de Santé Publique, AP-HP, Groupe hospitalier Albert Chenevier- Henri Mondor, F-94010 Créteil, France

**Keywords:** Electronic data collection, Costs, Time management, Work satisfaction

## Abstract

**Background:**

Electronic Case Report Forms (eCRFs) are increasingly chosen by investigators and sponsors of clinical research instead of the traditional pen-and-paper data collection (pCRFs). Previous studies suggested that eCRFs avoided mistakes, shortened the duration of clinical studies and reduced data collection costs.

**Methods:**

Our objectives were to describe and contrast both objective and subjective efficiency of pCRF and eCRF use in clinical studies. A total of 27 studies (11 eCRF, 16 pCRF) sponsored by the Paris hospital consortium, conducted and completed between 2001 and 2011 were included. Questionnaires were emailed to investigators of those studies, as well as clinical research associates and data managers working in Paris hospitals, soliciting their level of satisfaction and preferences for eCRFs and pCRFs. Mean costs and timeframes were compared using bootstrap methods, linear and logistic regression.

**Results:**

The total cost per patient was 374€ ±351 with eCRFs vs. 1,135€ ±1,234 with pCRFs. Time between the opening of the first center and the database lock was 31.7 months Q1 = 24.6; Q3 = 42.8 using eCRFs, vs. 39.8 months Q1 = 31.7; Q3 = 52.2 with pCRFs (p = 0.11). Electronic CRFs were globally preferred by all (31/72 vs. 15/72 for paper) for easier monitoring and improved data quality.

**Conclusions:**

This study found that eCRFs and pCRFs are used in studies with different patient numbers, center numbers and risk. The first ones are more advantageous in large, low–risk studies and gain support from a majority of stakeholders.

## Background

Collection of individual patient data on Case Report Forms (CRFs) in clinical research has traditionally been done by investigators in their offices summarizing medical charts on paper forms (pCRFs), a tedious method that could result in data errors and wrong conclusions [1,2]. Electronic data capture has in recent years been increasingly used in both industry and academic research settings [3,4]. The feasibility of electronic CRFs (eCRFs) has been documented by numerous studies analyzing data collected on websites, laptops or digital pens [5-10]. Since the mid-1980s, eCRFs have increased data quality and completeness by using alarms, automatic completions and reminders [11,12], reducing losses and transport logistics, especially for multicenter trials [13]. Moreover, use of eCRFs permits speedier database processing and shorter study periods, resulting in lower costs [7,11,14-19]. Previous studies of eCRFs have primarily focused on the investigators’ point of view, while few have documented the perspectives of the other stakeholders [5,6,20,21].

Despite their demonstrated usefulness, eCRFs have not become dominant [3]. Welker has identified some of the barriers to their dissemination: the lack of available on-site technology, insufficient assistance by Information Technologies staff or software providers, investigators’ lack of motivation and interference of eCRFs with their clinical tasks, complexity of installation and maintenance of the software and high investment cost [22]. The labor cost of data entry is transferred from clerks to investigators who may see few tangible benefits apart from a better quality of data and speedier study completion. While some investigators have integrated the electronic interface into their medical practice to the point of making it an asset [23], the majority views the implementation of an eCRF in a paper-based working environment as a source of redundancy [19,24].

Our objective was to formally describe the efficiency (measured by satisfaction, cost and duration of the study) of electronic and paper CRFs in the context of biomedical research conducted in hospitals.

## Methods

The primary endpoint was the satisfaction of stakeholders in a clinical study: investigators, clinical research associates (CRAs) and data managers (DMs). Secondary endpoints were costs and duration of the studies. Our hypotheses were that eCRFs save cost and time [18,22], and would be preferred by those 3 stakeholders [5].

### Material

#### Clinical studies: inclusion criteria

We retrospectively selected biomedical research studies monitored by 6 research units involved in eCRF testing, completed between 2007 and 2010 (or 2011 for eCRFs) and sponsored by the Paris regional hospital consortium AP-HP. The research topics were representative of the ongoing publicly funded clinical research. Paper (p) CRF studies were defined by the use of a CRF on paper, completed with a pen, and data entry by a data clerk. Electronic (e) CRF studies used computer data entry by the investigator or an assistant, online or offline. Two studies which used pCRFs to collect data before entering it in the eCRF were analyzed in the eCRF group.

#### Stakeholders

We investigated the satisfaction and preference of three stakeholder groups: investigators, clinical research associates (CRAs) and data managers (DMs) for both types of CRF. The investigators surveyed had included patients in the studies selected, belonged to 45 different hospitals and were primarily physicians or nurses; the CRAs and DMs were working for the Paris regional hospital consortium.

### Methods

#### Clinical studies

We collected protocols, budgets and expense statements, CRFs, monitoring reports and other relevant technical documents. Studies were characterized by phase, number of patients, purpose (therapeutic/ diagnostic/observational), geographical level and risk (from level A = minimal, e.g. trial involving only additional blood sample collection, to D = major risk, e.g. trial of innovative therapies, phase I or II trials). We estimated the duration of patients’ recruitment, the time between the opening of the first center and the database lock, and between the last visit of the last patient and the database lock (see Additional file 1).

#### Cost estimation

The cost of a study was estimated from: labor costs, i.e. expenditures for CRAs and DMs salaries during the study, and logistical costs, i.e. printing of paper CRFs, development of database and interface if done by an external company, cost of the eCRF, travel costs of CRAs and investigators, and randomization software. Since 2003, following a successful tender by TELEMEDICINE Technologies S.A.S., the AP-HP’s Department of Clinical Research and Development (*Direction de la Recherche Clinique et du Développement; DRCD*) has contracted with TELEMEDICINE for use of the software CleanWEB in eCRFs for clinical trials. In 2010, the global cost was 861.12€ for a trial with one or two centers and 9,687.60€ for three or more centers (1€ = US$1.33). We used the 2010 contract prices for the cost of each eCRF. Expenditures were updated to 2010 with the hypothesis that they were completed during the median year for studies lasting longer than a year. We estimated both the total cost and the cost per patient.

#### Stakeholders’ satisfaction

Satisfaction of the three stakeholder groups was measured through surveys (see Additional files 2, 3 and 4). Each stakeholder received their own questionnaire, consisting of demographics, closed-ended questions about satisfaction, preferences and self-reported usage patterns. The last part consisted of two open-ended questions to identify additional issues.

### Statistical analysis

#### Clinical studies

The unit of analysis was the study. Studies using pCRFs and eCRFs were compared by characteristics (phase, number of patients and risk), costs and timeframes. Fisher’s exact test was used for categorical variables and Wilcoxon sign-rank test for continuous ones. Due to the skewed distribution of costs and the sample size, costs per patient of pCRFs and eCRFs were compared using the bias-corrected and accelerated bootstrap (BCa) procedure with 2,000 replications to estimate the confidence interval [25]. We used a linear regression model to explain costs per patient and time between the opening of the first center and the freezing of the database. A log transformation was used to analyze costs per patient. The linear regression models included terms for data collection method (pCRF/eCRF), randomization, geographical area (regional/national), level of risk, number of patients, number of centers and number of variables, time from the opening of the first center to database lock. The regression models did not include the phase of the study which was highly correlated with patient numbers and would not have added extra information. A forward stepwise method was used, and the variable “pCRF/eCRF” was forced in the models. Finally, a logistic model was used to explain the choice of pCRF/eCRF. The model included randomization, geographical area, level of risk of the study, number of patients included, number of centers, number of variables, duration and number of medical teams involved [18].

#### Stakeholders’ satisfaction

The unit of analysis was the respondent. Descriptive statistics were performed on the answers of investigators, CRAs and DMs. Global satisfaction of stakeholders was compared using a Chi square test. Multinomial and basic logistic regression models were used to understand satisfaction and preferences, with the hypotheses that CRAs, DMs and youngest stakeholders should prefer eCRFs; models included type of stakeholder, age, sex, level of computer proficiency and research experience.

Answers to the two open-ended questions were classified by topic using word clustering for the question about key features of an optimal data collection form, and by topic clustering for the open remarks.

All analyses were performed using SAS® (Version 9.3 for Windows, SAS Institute, Inc., Cary NC, USA).

## Results

We included 27 clinical studies, 16 (59%) using pCRFs and 11 (41%) eCRFs.

The main characteristics of the studies are summarized in Table 1. Electronic CRFs studies were mostly large multicenter, national and phase 3 clinical trials while pCRFs studies were trials with few patients and centers. The majority of pCRFs were drug trials (15/16; p = 0.036), and eCRFs were more often used in trials with a significantly higher number of patients (355 vs. 60 patients in pCRFs; p = 0.014) and fewer data (17 vs. 39 pages with pCRFs; p = 0.027). The number of patients was the only explanatory variable for CRF choice (Table 2).

**Table 1:** Characteristics of the studies (n = 27)

		**pCRFs n = 16**	**eCRFs n = 11**	**All n = 27**
**Design**	**Non-interventional studies**	1 (6%)	4 (36.4%)	5 (18.5%)
	**Clinical trials**	15 (94%)	7 (63.6%)	22 (81.5%)
**Randomized**		10 (63%)	7 (64%)	17 (63%)
**Multicenter**		10 (63%)	9 (82%)	19 (70%)
**Geographic level**	**International/national**	7 (44%)	8 (73%)	15 (56%)
	**Regional**	9 (56%)	3 (27%)	12 (44%)
Purpose*	**Diagnostic**	1 (6%)	3 (27%)	4 (15%)
	**Observational**	0 (0%)	2 (18%)	2 (7%)
	**Therapeutic**	15 (94%)	6 (55%)	21 (78%)
**Risk level**^**α**^	**A**	2 (13%)	4 (36.4%)	6 (22%)
	**B**	4 (25%)	3 (27%)	7 (26%)
	**C**	3 (19%)	3 (27%)	6 (22%)
	**D**	7 (44%)	1 (9%)	8 (30%)
**Clinical trial phase**	**1**	1 (7%)	0 (0%)	1 (4%)
	**2**	4 (27%)	1 (14%)	5 (23%)
	**3**	7 (47%)	6 (86%)	13 (59%)
	**4**	3 (20%)	0 (0%)	3 (14%)
Median number of patients included*		60	355	80
		Q1 = 27	Q1 = 78	Q1 = 50
		Q3 = 141	Q3 = 700	Q3 = 500
**Median number of centers**		5	10	7
		Q1 = 1	Q1 = 6	Q1 = 1
		Q3 = 10	Q3 = 13	Q3 = 12
**Median planned duration of study (months)**		24	27	25
		Q1 = 16	Q1 = 18	Q1 = 16
		Q3 = 40	Q3 = 36	Q3 = 36
**Median planned patient follow up (days)**		137	60	91
		Q1 = 67	Q1 = 12	Q1 = 30
		Q3 = 365	Q3 = 112	Q3 = 213
**Median number of variables in CRF**		1,062	396	1011
		Q1 = 669	Q1 = 153	Q1 = 286
		Q3 = 1,118	Q3 = 1,567	Q3 = 1,126
**Median number of variables in database**^**β**^		65,928	304,929	76,692
		Q1 = 18,764	Q1 = 35,250	Q1 = 20,088
		Q3 = 171,646	Q3 = 625,865	Q3 = 304,929
Median number of full pages in CRF*		39	17	31
		Q1 = 28	Q1 = 9	Q1 = 17
		Q3 = 44	Q3 = 30	Q3 = 44

*Significant difference between eCRFs and pCRFs (*p* < 0.05).

^α^From level A = minimal, e.g. trial involving only additional blood sample collection, to D = major risk, e.g. trial of innovative therapies, phase I or II trials.

^β^Number of variables in database = number of patients x number of variables in CRF.

**Table 2:** Logistic regression model with data collection method as dependant variables (n = 27)

		**OR**	**IC**	**p**
**Geographic level**	**International/national**	1	-	0.14
	**Regional**	0.292	0.056 – 1.525	
**Risk level**	**A**	1	-	0.28
	**B**	0.375	0.039 – 3.605	
	**C**	0.500	0.049 – 5.154	
	**D**	0.071	0.005 – 1.059	
**Number of patients included**		1.004	1.000 – 1.009	0.04
**Number of centers**		1.004	0.963 – 1.047	0.85
**Number of variables**		0.999	0.998 – 1.000	0.23
**Planned duration of study**		0.987	0.928 – 1.050	0.68
**Number of medical teams involved**		0.936	0.369 – 2.374	0.89

The modeled probability is the choice of an eCRF for the study as opposed to a pCRF.

### Clinical studies

Time from the opening of the first center to database lock tended to be shorter with eCRFs (31.7 months vs. 39.8 months; p = 0.11). We found no difference in the average duration of recruitment (22.4 ±9 months with eCRFs vs. 26.5 ±13 months with pCRFs; p = 0.34) nor in time from the last visit of the last patient to database freeze (8 months with eCRFs vs. 8.8 months with pCRFs; p = 0.81). Linear regression found that the use of eCRF and the smaller number of centers were associated with shorter study durations (Table 3).

**Table 3:** Linear regression model with cost log and duration as dependant variables (n = 27)

		**Parameter estimate**	**SE**	***P *** **value**
**Duration of the study:**				
CRF*	**Paper**	-	-	0.045
	**Electronic**	−10.14	4.79	
Participating centers*		0.48	0.13	0.001
**Cost of the study (log):**				
**CRF**	**Paper**	-	-	0.41
	**Electronic**	0.29	0.35	
Design*	**Trial without randomization**	-	-	0.002
	**Trial with randomization**	−1.33	0.40	
	**Non-interventional study**	−2.51	0.62	
Number of patients included*		−0.001	0,0004	0.021

SE: standard error.

*Explanatory variable (*p* < 0.05).

**Figure 1:**
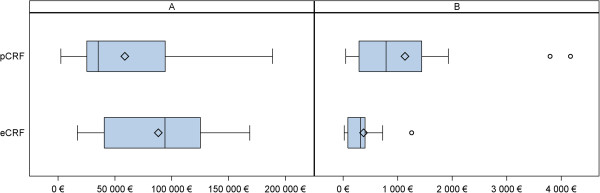
**Cost of the studies by data collection method. A**: Total cost; **B**: Total cost per patient.

The total average cost of a trial was higher with eCRFs (88,222€ ± 47,907) than with pCRFs (58,794€ ± 48,665), but the mean cost per patient was lower with eCRFs (374€ ± 351 vs. 1,135€ ± 1,234) (Figure 1). The difference in average cost per patient was 762€ per patient (95% CI bootstrap: [270€- 1613€]). Other explanatory variables were the design of the study – phase 1 and 2 trials without randomization being more expensive – and the number of patients included, but not the number of variables collected (Table 3).

### Stakeholders’ satisfaction

**Figure 2:**
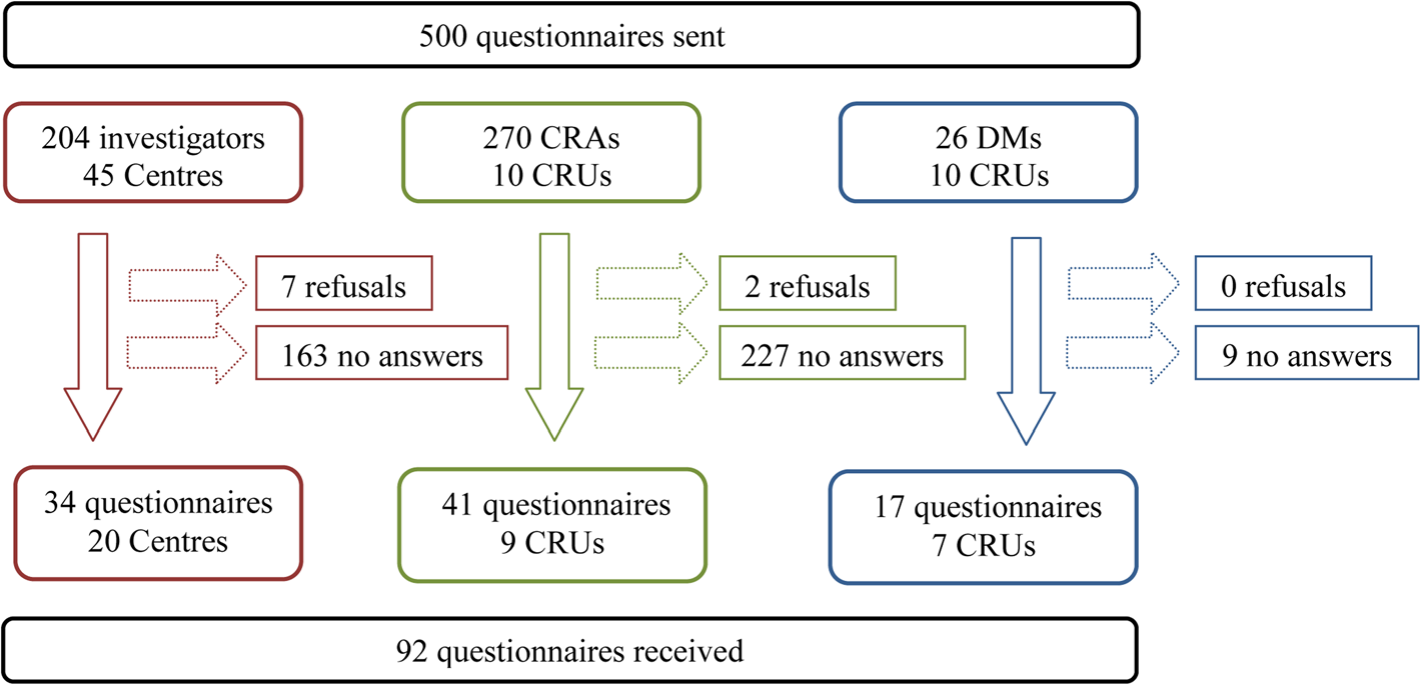
**Questionnaires flow-chart.** CRA = clinical research associate, DM = data manager, CRU = clinical research unit.

Thirty-four questionnaires were returned from investigators, including six from centers outside Paris. Forty-one questionnaires were returned from CRAs and 17 from DMs. Seven investigators declined to participate because of a lack of experience with eCRFs, and 2 CRAs because of a lack of time (Figure 2). Among the 34 answering investigators, 33 had experienced pCRFs, 29 eCRFs and 28 had experienced both and responded to comparison questions. The main characteristics of the respondents are summarized in Table 4.

**Table 4:** Characteristics of the respondents to the satisfaction and preference surveys

		**Investigators**	**CRAs**	**DMs**
**Respondent**		34	41	17
**Age**	**< 30**	0 (0%)	17 (42%)	7 (44%)
	**30 to 40**	8 (24%)	19 (46%)	7 (44%)
	**> 40**	26 (76%)	5 (12%)	2 (12%)
**Gender**	**M**	20 (59%)	6 (15%)	10 (59%)
	**F**	14 (41%)	35 (85%)	7 (41%)
**Computer proficiency level**	**Beginner**	1 (3%)	-	-
	**Average**	19 (56%)	-	-
	**Good**	14 (41%)	-	-
**Clinical research experience**	**< 1 year**	-	4 (10%)	2 (12%)
	**1 to 3 years**	-	19 (46%)	7 (41%)
	**3 to 5 years**	-	11 (27%)	3 (18%)
	**> 5 years**	-	7 (17%)	5 (29%)

CRA: clinical research associate, DM: data manager.

**Figure 3:**
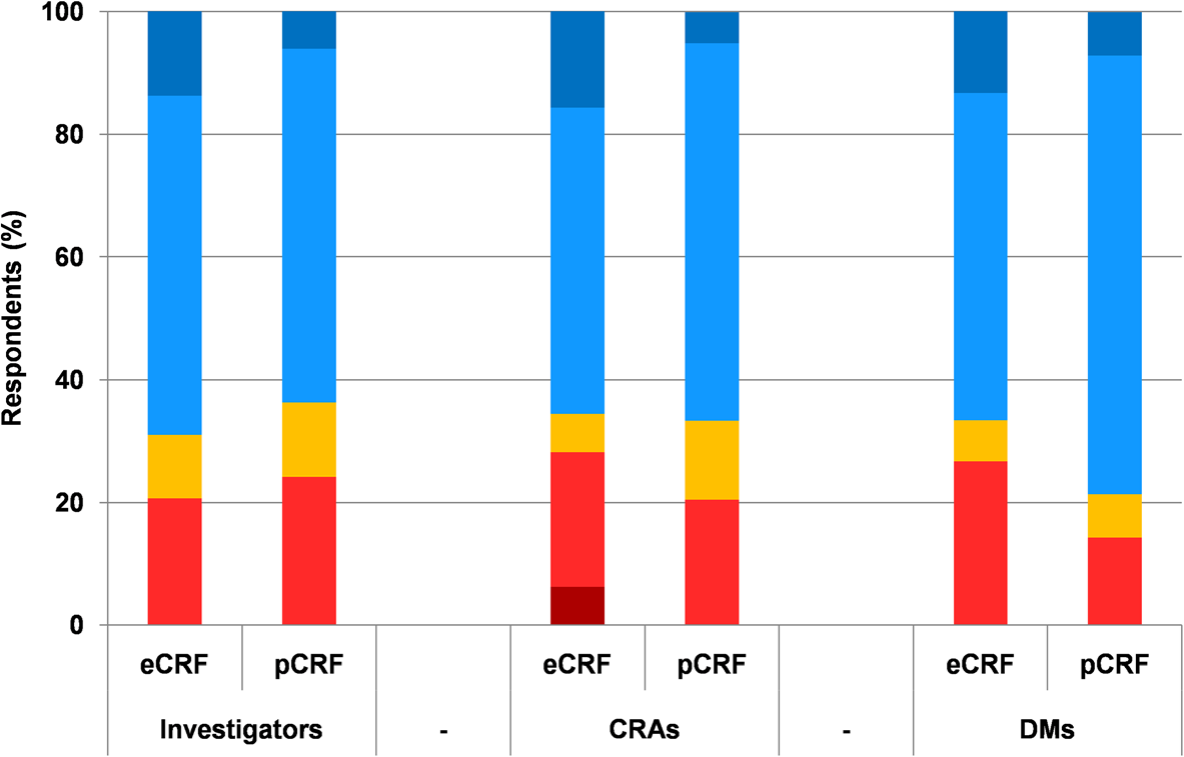
**Satisfaction of respondents regarding eCRF and pCRF data collection.** Percentage of satisfaction level for the 3 stakeholders (very satisfied: dark blue, fairly satisfied: light blue, no opinion: yellow, fairly unsatisfied: light red, very unsatisfied: dark red). CRA = clinical research associate, DM = data manager.

**Figure 4:**
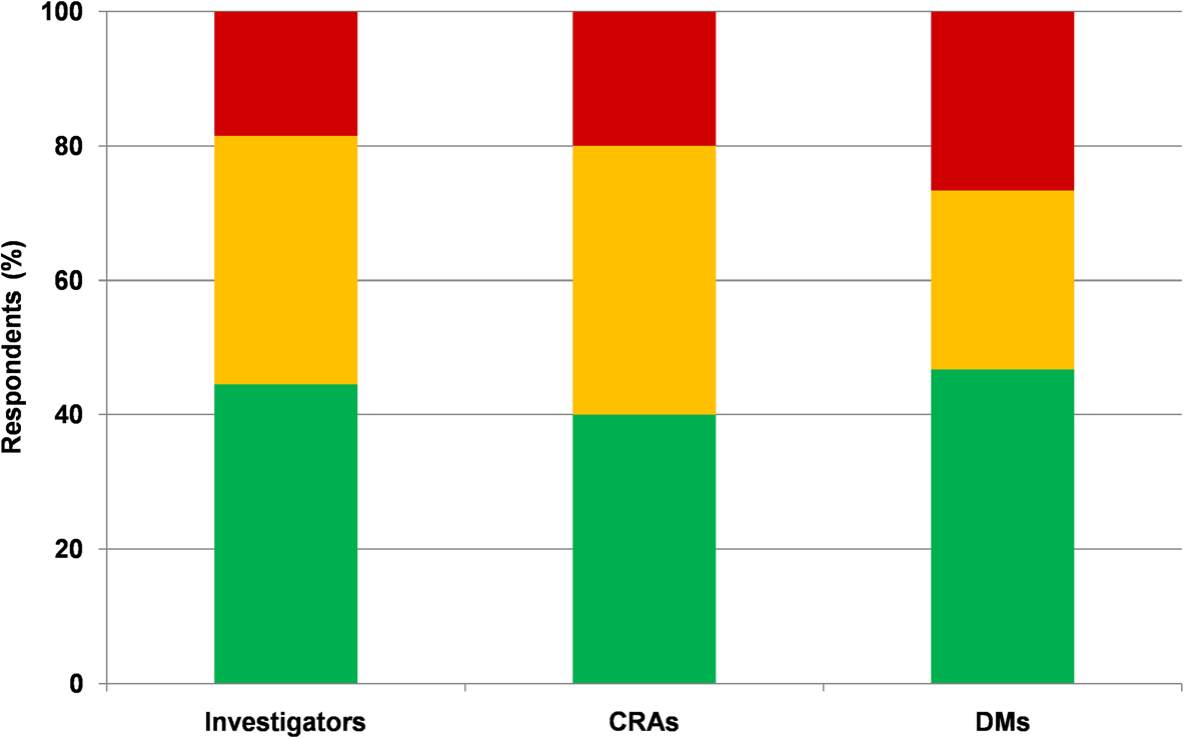
**Preferences of respondents between eCRF and pCRF data collection.** Percentage of stakeholders preferring pCRF (red), with no or mixed opinion (yellow) or preferring eCRF (green). CRA = clinical research associate, DM = data manager.

Overall, stakeholders were as satisfied with eCRFs as with pCRFs (51/76 vs. 58/86; p = 0.96) (Figure 3). When asked for their preference of one over the other, a majority of stakeholders chose eCRF (Figure 4).

Half of investigators (16/29) adapted to the eCRF user interface from the first patient. Half of them (15/29) experienced technical problems from time to time with eCRFs, and those were usually resolved within a day (23/29). One-third (10/29) was upset by the presence of immediate checks and constraints in the eCRF, while most (23/29) enjoyed the lack of constraints in a pCRF. Two-thirds of investigators (18/29) were satisfied with the intuitiveness of the eCRF interface. Twenty /29 investigators never entered data in the eCRF during consultations, while 15/33 did so when using a pCRF. Half (16/33) were satisfied that the pCRF did not affect or affected positively the patient-doctor relationship, while a majority (17/29) had no opinion on the impact of the eCRF. Data entry was found easier with pCRF (13/28 vs. 11/28). Nonetheless, investigators were more satisfied (24/29 vs. 9/33) with the logistics, storage and data safety of eCRFs. Finally, most investigators (24/29) would accept an eCRF in the future while only half (17/33) would use a pCRF.

Most CRAs preferred the eCRF (Figure 3), and one-third would choose a CRF depending on the trial characteristics. CRAs reported preference for pCRFs in monocentric trials including few patients while they would rather use an eCRF for a multicentric trial unless there were few patients and variables. Despite their preference for eCRFs, CRAs identified the following benefits of pCRFs: the greater acceptability by investigators, the tangibility of paper and the impetus to spend time in the centers to monitor the data collection. They based their preference for eCRFs on the more effective monitoring which allowed them to monitor data collection from their offices — for example by receiving queries of abnormal entries in real time—, the better prevention of errors resulting from it and from automatic checks, the easier electronic storage (as opposed to the copious paper storage of pCRFs) and a greater efficiency in managing drug supplies.

DMs preferred eCRFs (Figure 3) because fewer queries were generated and the database contained fewer errors before cleaning. Thus they saved time and allowed faster data availability.

No variable was associated significantly with satisfaction or preference. The following trends appeared: women (DMs and CRAs) were likely to prefer eCRF, younger and computer-proficient investigators were likely to be dissatisfied by eCRF, and stakeholders with a greater pCRF experience (>10 clinical studies) were likely to prefer pCRF (see Additional file 5 and Additional file 6).

The requirements of an optimal data collection are summarized in Table 5: *“eCRFs would be perfect at 100% if we could have the computer next to the patient”.* Additional issues are summarized in Table 6.

**Table 5:** “What are the key features of an optimal data collection method in a clinical study?”

	**Main features answered**	**Details**
**Investigators**	Quality interface (x23)	Fast, simple, without bugs and blocking, with flexible data entry
	Reliable data collection, with few queries (x8)	Alarms and mandatory fields
	Electronic format (x3)	Allowing data sharing without data recapture and duplication
**CRAs**	Quality interface (x24)	Fast, simple, without bugs and blocking, with flexible data entry
	Accessible form (x7)	
	Efficient monitoring (x5)	Real-time queries and remote consultations
	Quality of form (x5)	No free answers, short and clear questions and few variables
	Immediate controls during data entry (x3)	
	Motivation and availability of investigators (x3)	
**DMs**	Reliable data collection, few queries (x17)	Controls and constraints during data entry and queries emailed automatically
	Quality of database, easy to operate (x5)	
	Quality of form (x5)	Real-time queries and remote consultations
	Quality of form (x5)	No free answers, short and clear questions and few variables
	Quality interface (x3)	Ergonomic
	Maximum access fees and free action for data managers (x2)	

Responses from investigators, clinical research associates and data managers.

CRA: clinical research associate, DM: data manager.

**Table 6:** Main themes discussed by stakeholders in open-ended questions

Investigators	Complaints	About redundancy of data “It doesn’t matter whether it’s on paper or electronic, as long as data are entered only once."
		That some promoters want them to complete a pCRF first as source document and then to re-enter all the data in the eCRF.
		About software design companies “By trying to make money, firms that sell this type of CRF software developed templates that do not fit well with the variability of studies and data.”
	Hopes	To have their needs taken into account "eCRFs are developed by those who use data but never by those who enter the data and who have, in the present context, less and less availability.“
		Working with transportable computers “The graphic tablet, an eCRF transportable to the bedside, is the solution for future. It’s already used in anesthesia with great success.“
CRAs	Complaints	CTAs needed on site: “Whatever the collection method, investigators don’t have the time …”
		eCRF storage: CRFs still needs to be kept on paper, as source data and to be signed …
DMs	Complaints about CleanWEB consistency management	“Only simple checks can be defined in CleanWEB; more complicated ones must be programmed in SAS after database export.”
		Moreover, “the computer code managing the automatic controls should be accessible and easily understood."
		“The database structure isn’t known when designing eCRFs for CleanWEB, yet it is the first thing that must be established. And it’s currently impossible to have a structure that complies with CDISC."

Answers from investigators, clinical research associates and data managers.

CRA: clinical research associate, DM: data manager.

## Discussion

In this first description of the use of eCRFs and pCRFs across 27 clinical studies, we found that most stakeholders were satisfied with eCRFs and that the use of eCRFs was associated with shorter study duration and lower cost per patient. Average duration of recruitment did not differ between pCRF and eCRF studies, despite a greater number of trials investigating rare and pediatric conditions in the pCRF group.

Data managers reported that eCRFs saved time and improved data quality, however we cannot exclude that the perception of fewer queries simply results from a smaller number of variables monitored; clinical research associates valued the automated quality checks and easier storage of electronic data. Both DMs and CRAs preferred eCRF for multicenter trials. Improvements are needed to facilitate the integration of eCRFs into clinical practice, including widespread adoption of portable devices such as digital pens [6] or graphic tablets, which one investigator described as “*the solution for the future, and already used very successfully in anesthesia ”*.

It appeared that stakeholders’ characteristics do not predict preferences, except for young and computer literate investigators who tended to be more demanding with eCRFs, probably because they had greater expectations of eCRF and yet were more aware of its limitations.

In addition to exploring stakeholder perspectives, we found that eCRFs were mainly used in large, national and multicenter trials, whereas pCRFs were used in small high risk drug trials, possibly because of the greater reliability of written documents.

The few studies investigating stakeholders’ experiences of CRFs have mostly focused on investigators and found a high level of support for eCRFs [24]. We included two additional key stakeholders and revealed more mixed results in the level of satisfaction and preferences regarding eCRFs. CRAs agreed with the Litchfield hypothesis [5] that monitoring would be more efficient with an eCRF. Moreover, as expected [17,26], acceptance of eCRFs by investigators remained one of their biggest challenges. López-Carrero reported very positive reviews of eCRFs by investigators, with three-quarters finding data entry easier and more than a half saying that it decreased workload. We did not find investigators to be so optimistic about eCRF capacities, particularly with respect to data entry and workload. The fact that the López-Carrero [21] investigators were asked about a pilot project with a “specifically designed” eCRF may explain our less enthusiastic responses.

While literature reviews have found favorable results for eCRFs in terms of study duration and costs, we found that the lower cost per patient was explained by the large patient number in eCRF trials. This may be attributable to our sample which did not include pCRF studies with large patient numbers, or eCRF studies with small patient numbers and/or to the fact that most of the other cost studies were based on models [18] or used ad-hoc prototypes [27,28].

The retrospective non randomized design of our study was dictated by two major issues: time constraints and acceptability. Randomization would not have been acceptable to investigators who want to be able to make their own choices and it also would not have been able to show that eCRF and pCRF have their specific indications and are not therefore perfect substitutes. There is a kind of ‘indication bias’ as shown both in Table 1 and in the stakeholders’ responses. Indeed we have shown that for some studies pCRF is cheaper and appears more trustworthy. In addition, unobserved factors must also influence the choice between eCRF and pCRF as can be assumed by, for example, the fact that the large difference in the number of variables between the 2 collection methods was not correlated to any of the characteristics of the studies that we investigated.

Whilst the CRA and Investigator questionnaires response rates were low, due to many stakeholders having changed institutions and no longer being locatable, they emanated from 9/10 AP-HP units and 20 different hospitals spread over the French territory. Our small sample of respondents is mirrored in other studies, López-Carrero et al. [21] had a similar problem with only 27 investigators answering from 33 centers, and Lium et al. [24] interviewed 18 physicians, but from only 2 centers.

Most clinical trials sponsored by the AP-HP currently use CleanWEB for eCRFs. As a result, the stakeholders’ answers were related to that software. Our results may not adequately reflect the current situation of eCRF since half of the studies using eCRFs had started between 2004 and 2006 and are not representative of the current capacities of electronic data capture. Recent adaptations of CleanWEB include certification with Clinical Data Interchange Standards Consortium CDISC^a^, the possibility to connect to the server on-line or enter data off-line and direct data export to SAS, which may improve future satisfaction with eCRFs.

We were unable to suggest a financial advantage in using eCRFs in trials with fewer than 50 patients, given that the smallest eCRF trial had 47 patients. Nonetheless, CleanWEB price discounts for monocentric eCRFs could make it cost-efficient, even for small trials. Likewise, we were not able to draw any conclusions regarding pCRF trials with more than 700 patients. We did not take into account the annual maintenance package of 125,580€, billed to the institution for all studies using CleanWEB. We used bootstrap replications for the cost analysis because of the small number of studies and because calculation of means after log transformation resulted in a comparison of geometric mean costs instead of arithmetic means. The bootstrap method requires the true distribution of the data to be adequately represented by its empirical distribution [25]. Finally, as we did not select the studies based on their characteristics, we had much heterogeneity in terms of risk level and trial phase.

An important aspect in comparing pCRFs and eCRFs that we have only touched upon in our questionnaires is the management of the delivery of drugs and placebo in pharmaceutical trials. Computerized treatment management programs that may be included in eCRF software enable investigators to streamline complex processes, including: managing the supply of medication to the centers even for long-term treatments, managing intricate protocols and overseeing product expirations. This system manages stock control and prevents waste, thus preventing breaks in inclusions due to supply ruptures and avoiding mistakes in treatment delivery to the patient.

## Conclusion

This study examined eCRFs and pCRFs from the viewpoint of investigators and other important stakeholders. It found that eCRFs and pCRFs are used in studies with different patient numbers, center numbers and risk. The first ones are more advantageous in large, low–risk studies and gain support from a majority of stakeholders. Our findings also suggest that eCRF and pCRF may not be substitutes but complement each other with their own specific indications. The choice between paper and electronic CRF is a significant step in the design and execution of clinical studies; it should be discussed with the involved stakeholders and based on efficiency.

## Endnote

^a^CDISC: standards of acquisition, exchange, submission and archive of clinical research data that enable information system interoperability to improve medical research.

## Abbreviations

eCRF: Electronic case report form; pCRF: Paper case report form; CRAs: Clinical research associates; DM: Data managers; AP-HP: Assistance Publique-Hôpitaux de Paris; CDISC: Clinical data interchange standards consortium.

## Competing interests

The authors declare that they have no competing interests.

## Authors’ contributions

ALJ, CA and IDZ conceived and designed the experiment. ALJ performed the experiment. ALJ and CQ realized the acquisition and management of data, and analyzed the data. ALJ, CQ and IDZ wrote the paper. All authors read, commented and approved the final manuscript.

## Pre-publication history

The pre-publication history for this paper can be accessed here:

http://www.biomedcentral.com/1471-2288/14/7/prepub

## Supplementary Material

Additional file 1 Data collection form regarding studies’ characteristics, variables and costs. Click here for file

Additional file 2 Satisfaction questionnaire addressed to investigators. Click here for file

Additional file 3 Satisfaction questionnaire addressed to clinical research associates. Click here for file

Additional file 4 Satisfaction questionnaire addressed to data managers. Click here for file

Additional file 5 Analysis of investigators’ satisfaction and preferences. Click here for file

Additional file 6 Analysis of CRAs and DMs’ satisfaction and preferences. Click here for file

## The CompaRec investigators

CRUs

P. Maison (AP-HP, CHU Henri Mondor, Unité de Recherche Clinique-Paris Est-Mondor, F-94010 Créteil, France), R. Serreau (AP-HP, Hôpital Saint Antoine, Unité de Recherche Clinique-Paris Est, F-75012 Paris, France), T. Simon (AP-HP, Hôpital Saint Antoine, Unité de Recherche Clinique-Paris Est, F-75012 Paris, France), P. Aegerter (AP-HP, Hôpital Ambroise Paré, Unité de Recherche Clinique-Paris Île-de-France Ouest, F-92100 Boulogne, France), F. Tubach (AP-HP, Groupe hospitalier Bichat-Claude Bernard, Unité de Recherche Clinique-Paris Nord Val de Seine, F-75018 Paris, France), G. Chatellier (AP-HP,Hôpital Européen Georges Pompidou, Unité de Recherche Clinique-Paris Ouest, F-75015 Paris, France), L. Becquemont (AP-HP, Hôpital Bicêtre, Unité de Recherche Clinique- Paris Sud, F-94275 Le Kremlin-Bicêtre, France), A. Mallet (AP-HP, Hôpital de la Pitié-Salpêtrière, Unité de Recherche Clinique-Pitié Salpêtrière-Charles Foix, F-75013 Paris, France) and F. Maugard (AP-HP, Hôpital Saint Louis, DIRC/DRCD, F-75010 Paris, France);

Investigators

R. Yiou (AP-HP, CHU Henri Mondor, Service d’Urologie, F-94000 Créteil, France), B. Godeau (AP-HP, CHU Henri Mondor, Service de Médecine Interne, F-94000 Créteil, France), P. Cesaro (AP-HP, CHU Henri Mondor, Service de Neurologie, F-94000 Créteil, France), A. Mekontso Dessap (AP-HP, CHU Henri Mondor, Service de Réanimation médicale, F-94000 Créteil, France), O. Bouillanne (AP-HP, Hôpital Émile-Roux, Service de Gérontologie, F-94450 Limeil-Brévannes, France), G. Bobrie (AP-HP,Hôpital Européen Georges Pompidou, Service d’Hypertension Arterielle, F-75015 Paris, France), C. Faisy (AP-HP,Hôpital Européen Georges Pompidou, Service de Réanimation Médicale, F-75015 Paris, France), L. Bouadma (AP-HP, Groupe Hospitalier Bichat-Claude-Bernard, Service de Réanimation Médicale et Infectieuse, F-75018 Paris, France), S. Legrain (AP-HP, Groupe Hospitalier Bichat-Claude-Bernard, Service de Gériatrie, F-75018 Paris, France), F. Degos (AP-HP, Hôpital Beaujon, Service d’Hépatologie, F-92110 Clichy, France), D. Annane (AP-HP, Hôpital Pointcarré, Service de Réanimation Médicale, F-75010 Paris, France), B. Schlemmer (AP-HP, Hôpital Saint Louis, Service de Réanimation Médico-chirurgicale, F-92380 Garches, France), E. Thervet (AP-HP, CHU Necker-Enfants Malades, Service de Transplantation Rénale, F-75015 Paris, France ), R. Hankard (AP-HP, Hôpital Robert Debré, Comité de Liaison Alimentation Nutrition (CLAN), F-75019 Paris, France), C. Loirat (AP-HP, Hôpital Robert Debré, Service de Néphrologie Pédiatrique, F-75019 Paris, France), D. Bremond-Gignac (AP-HP, Hôpital Robert Debré, Service d’Ophtalmologie Pédiatrique, F-75019 Paris, France), N. Beydon (AP-HP, hôpital Armand-Trousseau, Unité fonctionnelle d'explorations fonctionnelles respiratoires, F-75012 Paris, France), E. Konofal (AP-HP, Hôpital Robert Debré, Centre pédiatrique des pathologies du sommeil, F-75019 Paris, France), Y.E. Claessens (Centre Hospitalier Princesse Grace, Département de Médecine d’Urgence, MC-98012 Monaco), G. Princ (AP-HP, Groupe Hospitalier Cochin-Saint-Vincent-de-Paul, Service de Stomatologie, F-75014 Paris, France), A.S. Rigaud (AP-HP, Hôpital Broca, Service de Gérontologie, F-75013 Paris, France), G. Cheron (AP-HP, CHU Necker-Enfants Malades, Service des urgences pédiatriques, F-75015 Paris, France), D. Joly (AP-HP, CHU Necker-Enfants Malades, Service de Nephrologie, F-75015 Paris, France), L. Guillevin (AP-HP, Groupe Hospitalier Cochin-Saint-Vincent-de-Paul, Service de médecine interne et Centre de Référence Maladies Rares, F-75014 Paris, France), N. Costedoat-Chalumeau (AP-HP, Hôpital de la Pitié-Salpêtrière, Service de Médecine Interne, F-75013 Paris, France) and D. Thabut (AP-HP, Hôpital de la Pitié-Salpêtrière, Service d’Hépato-Gastroentérologie, F-75013 Paris, France).
