# Supplementary material for: Comparison of two data collection processes in clinical studies: electronic and paper case report forms
Source: BMC Med Res Methodol. 2014 Jan 17;14:7. doi: 10.1186/1471-2288-14-7 (PMC3909932; doi:10.1186/1471-2288-14-7)
Supplement: Additional file 2 — Satisfaction questionnaire addressed to investigators. [file 1471-2288-14-7-S2.doc]

**
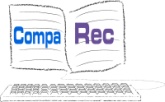
**
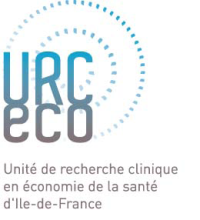
**CompaRec: Questionnaire for Investigators**

**Compared data collection methods:**

**Paper CRF (pCRF): the** classic method, in which the form is completed on paper by the investigator. Data are then entered in the database by 1 or 2 data clerks.

**Electronic CRF (eCRF):** data are directly entered by the investigator into a computerized CRF, which may be connected to the database via internet (computer, digital notepad, digital pen…). For the AP-HP research units this is usually CleanWeb.

**A- PERSONAL DETAILS**

1. **Age:**

< 30 years old 30 - 40 years old 40 - 50 years old  > 50 years old

1. **Gender:**

Female  Male

1. **Current position:**

Academic

Non-academic

Researcher

Specify:

1. **Specialty:**

Medical

Surgical

Obstetric

Specify:

1. **Have you already had experience as the primary investigator of a study?**

Yes  No

1. **Current computer proficiency level:**

Beginner

Average

Good

1. **In how many studies have you participated in the past 5 years?**
2. **Using pCRF:** 0  1 to 5  6 to 10  11 to 20  >20

1. **Using eCRF:**  0  1 to 5  6 to 10  >10

**B- YOUR OPINIONS ABOUT THE PAPER CRF DATA COLLECTION METHOD**

1. **Have you already participated in a study using a paper CRF?**

Yes  No

1. **If no, what is your opinion about this collection method?**

Bad

Good

No opinion

*If you have experience with pCRF data collection, go to question 3; if not, go directly to part C page 4.*

1. **If you have experience with pCRF data collection, how would you characterize your overall satisfaction with this method?**

Very unsatisfied  Fairly unsatisfied  No opinion  Fairly satisfied  Very satisfied

1. **a. In general, when you completed the pCRF, would you do it in front of the patient?**

Never  Rarely  Often  Always

1. **What are your opinions about using pCRFs in terms of the following:**

|  | **The pCRF proved to be:** | |  |
| --- | --- | --- | --- |
|  | **Well suited** | **Embarrassing** | **No opinion** |
| • The place of clinical practice (bedside, office…) |  |  |  |
| • Relationship with the patient |  |  |  |
| • Time management |  |  |  |

1. **If you did not complete the pCRF directly in front of the patient, when would you usually do so?**

Immediately  Within 24h  In 24-48h  In 48h-1wk

1 wk or more later  Never, a nurse did this for me

1. **What is your opinion about the ease of completing a pCRF?**

Fairly easy

Fairly difficult

1. **What do you think about the pCRF with respect to:**

|  | **1**  **Very unsatisfied** | **2**  **Unsatisfied** | **3**  **No opinion** | **4**  **Satisfied** | **5**  **Very satisfied** | **NA*** |
| --- | --- | --- | --- | --- | --- | --- |
| • The relative freedom of completion and correction |  |  |  |  |  |  |
| • The integration of pCRF into the organization of your clinical practice |  |  |  |  |  |  |
| • Its impact on the doctor-patient relationship |  |  |  |  |  |  |
| • The presentation of this method(ease of browsing, carrying) |  |  |  |  |  |  |
| • The completion speed of the pCRF |  |  |  |  |  |  |
| • The ease of immediate correction of an error |  |  |  |  |  |  |
| • The ease of resolving queries **^a^** |  |  |  |  |  |  |
| • The quality of the data collected |  |  |  |  |  |  |
| • Medical staff logistics **^b^** |  |  |  |  |  |  |
| • Backup, storage and data security |  |  |  |  |  |  |
| • The cost of the study |  |  |  |  |  |  |
| • Other (specify): |  |  |  |  |  |  |

***** Not Applicable.

**^a^** Query = report for errors or missing data.

**^b^** Coordination of medical staff involved in CRF completion during the study.

1. **If you had choice, would you use the pCRF data collection method for a future study?**

Yes No

**C- YOUR OPINIONS ABOUT THE ELECTRONIC CRF DATA COLLECTION METHOD**

1. **Have you already participated in a study using an eCRF?**

Yes  No

1. **If no, what is your opinion about this method?**

Bad

Good

No opinion

*If you have experiment with the eCRF data collection, go to question 3, if not, go directly to part E page 9.*

1. **If you have experience with eCRF data collection, how would you characterize your overall satisfaction?**

Very unsatisfied  Fairly unsatisfied  No opinion  Fairly satisfied  Very satisfied

1. **a. In general, when you completed the eCRF, would you do it in front of the patient?**

Never  Rarely  Often  Always

1. **What are your opinions about using eCRFs in terms of the following:**

|  | **The eCRF proved to be :** | |  |
| --- | --- | --- | --- |
|  | **Well-suited** | **Embarrassing** | **No opinion** |
| • The place of clinical practice (bedside, office…) |  |  |  |
| • Relationship with the patient |  |  |  |
| • Time management |  |  |  |

1. **If you did not complete the eCRF directly in front of the patient, when would you usually do so?**

Immediately  Within 24h  In 24-48h  In 48h-1wk

1 wk or more later  Never, a nurse did this for me

1. **What is your opinion about the ease of completing an eCRF?**

Fairly easy

Fairly difficult

1. **How quickly did you adapt to using the electronic interface?**

From the first patient

After few patients

After many patients

1. **a. Did you have any technical problems linked to the use of the eCRF?**

Never **go to question 9**

Rarely

Sometimes

Often

**Go to question 8b**

**b. What kind of problems did you usually encounter?**

|  | **Yes** | **No** |
| --- | --- | --- |
| • Log in problems |  |  |
| • Page scroll too slow |  |  |
| • Bugs resulting in loss of unsaved data |  |  |
| • Blocked by the form completion constraints |  |  |

**c. Did these problems ever embarrass you in your work?**

Not at all

A little

Moderately

A lot

**d. In general, how long would it take for the problem to be resolved?**

Immediately

Within the day

Never

1. **What do you think about the eCRF with respect to:**

|  | **1**  **Very unsatisfied** | **2**  **Unsatisfied** | **3**  **No opinion** | **4**  **Satisfied** | **5**  **Very satisfied** | **NA** |
| --- | --- | --- | --- | --- | --- | --- |
| • The presence of immediate checks and constraints **^c^** |  |  |  |  |  |  |
| • The integration of eCRF in the organization of your clinical practice |  |  |  |  |  |  |
| • Its impact on the doctor-patient relationship |  |  |  |  |  |  |
| • The intuitiveness of the interface |  |  |  |  |  |  |
| • The completion speed of the pCRF |  |  |  |  |  |  |
| • The ease of immediate correction of an error |  |  |  |  |  |  |
| • The ease of resolving queries **^a^** |  |  |  |  |  |  |
| • The quality of data collected |  |  |  |  |  |  |
| • Medical staff logistics **^b^** |  |  |  |  |  |  |
| • Technical problems |  |  |  |  |  |  |
| • Backup, storage and data security |  |  |  |  |  |  |
| • The cost of the study |  |  |  |  |  |  |
| • Other (specify): |  |  |  |  |  |  |

***** Not Applicable.

**^a^** Query = report for errors or missing data.

**^b^** Coordination of medical staff involved in CRF filling during the study.

**^c^** When completing the eCRF, automatic controls may block the entry or alert the user when an entry is not consistent with the expected data type (date, text, volume ...)

1. **If you had choice, would you use the eCRF data collection method for a future study?**

Yes No

**D- COMPARISONS OF THE TWO DATA COLLECTION METHODS**

**If you have experience with both data collection methods, eCRFs and pCRFs:**

1. **For each of the following, do you prefer the pCRF, the eCRF or either of the two?**

|  | **pCRF** | **eCRF** | | **No opinion** | | **NA** |
| --- | --- | --- | --- | --- | --- | --- |
| • The number of errors when completing |  |  | |  | |  |
| • The completion itself (speed, ease of use) |  |  | |  | |  |
| • Query resolution |  |  | |  | |  |
| • The immediate correction of an error |  |  | |  | |  |
| • Resupplying of treatments |  |  |  | |  | |
| • Freedom in data entry |  |  |  | |  | |
| • Integration into clinical practice |  |  |  | |  | |

1. **Finally, of both data collection methods, which one do you prefer?**

eCRF  pCRF No opinion

**E- AND MORE GLOBALLY…**

1. **You generally participate in a study because you would like:**

|  | **Not at all** | **A little** | **Very much** |
| --- | --- | --- | --- |
| • Standardize health care protocols in your specialty |  |  |  |
| • Improve care in your specialty |  |  |  |
| • Enhance your work |  |  |  |
| • Develop interprofessional relationships |  |  |  |
| • Earn additional income |  |  |  |
| • Professional development opportunities |  |  |  |

1. **In your opinion, what would be, in order of importance, the key features of an optimal data collection method in a clinical study?**


5. **If you wish to address a topic not covered in this questionnaire, but which you think is important about these two modes of data collection in a clinical trial, please note it, explaining your reasoning:**

|  |
| --- |
