# Supplementary material for: Comparison of two data collection processes in clinical studies: electronic and paper case report forms
Source: BMC Med Res Methodol. 2014 Jan 17;14:7. doi: 10.1186/1471-2288-14-7 (PMC3909932; doi:10.1186/1471-2288-14-7)
Supplement: Additional file 3 — Satisfaction questionnaire addressed to clinical research associates. [file 1471-2288-14-7-S3.doc]

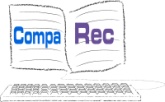

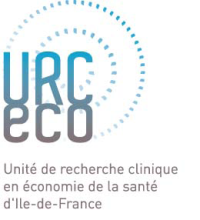
**CompaRec: Questionnaire**

**Data Managers**

**Compared data collection methods:**

**Paper CRF (pCRF): the** classic method, in which the form is completed on paper by the investigator. Data are then entered in the database by 1 or 2 data clerks.

**Electronic CRF (eCRF):** data are directly entered by the investigator into a computerized CRF, which may be connected to the database via internet (computer, digital notepad, digital pen…). For the AP-HP research units this is usually CleanWeb.

**A- PERSONAL DETAILS**

1. **Age:**

< 30 years old 30 - 40 years old >40 years old

1. **Gender:**

Female  Male

1. **How many years of experience in clinical research?**

< 1 years 1 - 3 years 3 - 5 years > 5 years

1. **In how many studies have you participated?**
2. **Using pCRF:** 0 1  2  3  4  5 6 à 10  >10

1. **Using eCRF:** 0 1  2  3  4  5 6 à 10  >10
2. **Using both methods at the same time:** 0 1  2  3  4  5 6 à 10  >10

**B- YOUR OPINIONS ABOUT THE ELECTRONIC CRF DATA COLLECTION METHOD**

1. **If you have experience with eCRF data collection, what is your overall satisfaction with the software you have used?**

|  | **1**  **Very unsatisfied** | **2**  **Unsatisfied** | **3**  **No opinion** | **4**  **Satisfied** | **5**  **Very satisfied** | **NA*** |
| --- | --- | --- | --- | --- | --- | --- |
| • CleanWEB |  |  |  |  |  |  |
| • Other (specify): |  |  |  |  |  |  |
| • Other (specify): |  |  |  |  |  |  |

* Not applicable.

1. **Which software are you using?**

CleanWEB

Other (specify):

1. **If you are using different database creation software, which one do you prefer?**

CleanWEB

Other (specify):

No preference

1. **If you prefer a particular software, what is/are the reason(s)?**

|  | **Yes** | **No** | **NA** |
| --- | --- | --- | --- |
| • Faster and easier creation of the database and interface |  |  |  |
| • Permits more freedom in programming |  |  |  |
| • Easier generation and processing of queries |  |  |  |
| • Easier operation of database |  |  |  |
| • Other (specify): |  |  |  |

1. **And what do you think more precisely of eCRF:**
2. **If you are working with CleanWEB:**

|  | **1**  **Very unsatisfied** | **2**  **Unsatisfied** | **3**  **No opinion** | **4**  **Satisfied** | **5**  **Very satisfied** | **NA** |
| --- | --- | --- | --- | --- | --- | --- |
| • The workload given the timeframe |  |  |  |  |  |  |
| • The use of your qualifications |  |  |  |  |  |  |
| • The ease of database and interface creation |  |  |  |  |  |  |
| • The quality of data before database cleaning |  |  |  |  |  |  |
| • The ease of database exploitation ^a^ |  |  |  |  |  |  |
| • Traceability of information |  |  |  |  |  |  |
| • Other (specify): |  |  |  |  |  |  |

^a^ database better built, easier to generate queries or manage a problem.

1. **If you are using software other than CleanWEB :**

|  | **1**  **Very unsatisfied** | **2**  **Unsatisfied** | **3**  **No opinion** | **4**  **Satisfied** | **5**  **Very satisfied** | **NA** |
| --- | --- | --- | --- | --- | --- | --- |
| • The workload given the timeframe |  |  |  |  |  |  |
| • The use of your qualifications |  |  |  |  |  |  |
| • The ease of database and interface creation |  |  |  |  |  |  |
| • The quality of data before database cleaning |  |  |  |  |  |  |
| • The ease of database exploitation ^a^ |  |  |  |  |  |  |
| • Traceability of information |  |  |  |  |  |  |
| • Other (specify): |  |  |  |  |  |  |

^a^ database better built, easier to generate queries or manage a problem.

**C- YOUR OPINIONS ABOUT THE PAPER CRF DATA COLLECTION METHOD**

1. **a. If you have experience with pCRF data collection, is data entry usually done in you unit?**

Yes  No

1. **If yes, how would you characterize your overall satisfaction with this method, depending on the software you have used:**

|  | **1**  **Very unsatisfied** | **2**  **Unsatisfied** | **3**  **No opinion** | **4**  **Satisfied** | **5**  **Very satisfied** | **NA** |
| --- | --- | --- | --- | --- | --- | --- |
| • Access |  |  |  |  |  |  |
| • Other (specify): |  |  |  |  |  |  |
| • Other (specify): |  |  |  |  |  |  |

1. **If your are working with different software, which one do you prefer?**

Access

Other (specify):

No preference

1. **If you prefer one of them, what is (are) the reason(s)?**

|  | **Yes** | **No** | **NA** |
| --- | --- | --- | --- |
| • Faster and easier creation of the database and interface |  |  |  |
| • Permits more freedom in programming |  |  |  |
| • Easier generation and processing of queries |  |  |  |
| • Easier database operation |  |  |  |
| • Other (specify): |  |  |  |

1. **And what do you think more precisely of pCRF:**
2. **If you are working with Access :**

|  | **1**  **Very unsatisfied** | **2**  **Unsatisfied** | **3**  **No opinion** | **4**  **Satisfied** | **5**  **Very satisfied** | **NA** |
| --- | --- | --- | --- | --- | --- | --- |
| • The workload given the timeframe |  |  |  |  |  |  |
| • The use of your qualifications |  |  |  |  |  |  |
| • The ease of database and interface creation |  |  |  |  |  |  |
| • The quality of data before database cleaning |  |  |  |  |  |  |
| • The ease of database exploitation ^a^ |  |  |  |  |  |  |
| • Traceability of information |  |  |  |  |  |  |
| • Other (specify): |  |  |  |  |  |  |

^a^ database better built, easier to generate queries or manage a problem.

1. **If you are using an other software than Access :**

|  | **1**  **Very unsatisfied** | **2**  **Unsatisfied** | **3**  **No opinion** | **4**  **Satisfied** | **5**  **Very satisfied** | **NA** |
| --- | --- | --- | --- | --- | --- | --- |
| • The workload given the timeframe |  |  |  |  |  |  |
| • The use of your qualifications |  |  |  |  |  |  |
| • The ease of database and interface creation |  |  |  |  |  |  |
| • The quality of data before database cleaning |  |  |  |  |  |  |
| • The ease of database exploitation ^a^ |  |  |  |  |  |  |
| • Traceability of information |  |  |  |  |  |  |
| • Other (specify): |  |  |  |  |  |  |

**D- COMPARISONS OF THE TWO DATA COLLECTION METHODS**

1. **a. Software that your team usually uses to create pCRF databases:**

**b. Software that your team usually uses to create eCRF databases:**

*Respond to the following questions based on the software that you usually use.*

1. **a. If you have experience with both data collection methods, eCRFs and pCRFs, which one do you prefer?**

eCRF  pCRF No opinion Depends on the situation

1. **If you prefer the eCRF, what are the reasons?**

|  | **Yes** | **No** | **NA** |
| --- | --- | --- | --- |
| • Security of immediate checks and constraints during completion |  |  |  |
| • The faster availability of data (intermediate analysis) |  |  |  |
| • The possibility to adjust the CRF continuously during the study |  |  |  |
| • The better traceability of information |  |  |  |
| • The database is easier to use |  |  |  |
| • The lowest rate of queries |  |  |  |
| • Management of electronic data only |  |  |  |
| • The time savings |  |  |  |
| • Other (specify): |  |  |  |

1. **If you prefer pCRFs, what are the reasons?**

|  | **Oui** | **Non** | **NA** |
| --- | --- | --- | --- |
| • No technical problems |  |  |  |
| • Better traceability of information |  |  |  |
| • The database is easier to use |  |  |  |
| • The lowest rate of queries |  |  |  |
| • The time savings |  |  |  |
| • Other (specify): |  |  |  |

1. **If your opinion depends upon the situation, which method would you prefer for each type of study:**

|  | **eCRF** | **pCRF** | **No opinion** |
| --- | --- | --- | --- |
| • Monocentric studies, few patients, few variables |  |  |  |
| • Monocentric studies, few patients, many variables |  |  |  |
| • Monocentric studies, many patients, few variables |  |  |  |
| • Monocentric studies, many patients, many variables |  |  |  |
| • Multicentric studies, few patients, few variables |  |  |  |
| • Multicentric studies, few patients, many variables |  |  |  |
| • Multicentric studies, many patients, few variables |  |  |  |
| • Multicentric studies, many patients, many variables |  |  |  |

**Based on your responses in the above table:**

**If you think the number of patients is important, what do you think is the limit?**

      patients.

**If you think the number of variables is important, what do you think is the limit?**

      variables.

**If you prefer the eCRF for multicentric studies, how many centers must there be to make it worthwhile?**

      centers.

**E- OPEN-ENDED RESPONSES**

1. **In your opinion, what would be, in order of importance, the key features of an optimal data collection method in a clinical study?**


5. **If you wish to address a topic not covered in this questionnaire, but which you think is important about these two methods of data collection in a clinical trial, please note it, explaining your reasoning:**

|  |
| --- |
