# Supplementary material for: Comparison of two data collection processes in clinical studies: electronic and paper case report forms
Source: BMC Med Res Methodol. 2014 Jan 17;14:7. doi: 10.1186/1471-2288-14-7 (PMC3909932; doi:10.1186/1471-2288-14-7)
Supplement: Additional file 4 — Satisfaction questionnaire addressed to data managers. [file 1471-2288-14-7-S4.doc]

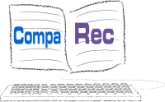

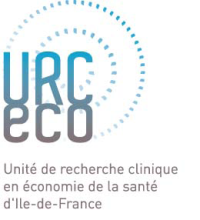
**CompaRec: Questionnaire for**

**Research Clinical Associates**

**Compared data collection methods:**

**Paper CRF (pCRF): the** classic method, in which the form is completed on paper by the investigator. Data are then entered in the database by 1 or 2 data clerks.

**Electronic CRF (eCRF):** data are directly entered by the investigator into a computerized CRF, which may be connected to the database via internet (computer, digital notepad, digital pen…). For the AP-HP research units this is usually CleanWeb.

**A- PERSONAL DETAILS**

1. **Age:**

< 30 years old 30 - 40 years old >40 years old

1. **Gender:**

Female  Male

1. **How many years of experience in clinical research?**

< 1 years 1 - 3 years 3 - 5 years > 5 years

1. **In how many studies have you participated?**
2. **Using pCRF:** 0 1  2  3  4  5 6 à 10  >10

1. **Using eCRF:** 0 1  2  3  4  5 6 à 10  >10
2. **Using both methods at the same time:** 0 1  2  3  4  5 6 à 10  >10

**B- YOUR OPINIONS ABOUT THE ELECTRONIC CRF DATA COLLECTION METHOD**

1. **If you have experience with eCRF data collection, how would you characterize your overall satisfaction?**

Very unsatisfied  Fairly unsatisfied  No opinion  Fairly satisfied  Very satisfied

1. **a. What do you think more precisely of eCRF?**

| With respect to: | **1**  **Very unsatisfied** | **2**  **Unsatisfied** | **3**  **No opinion** | **4**  **Satisfied** | **5**  **Very satisfied** | **NA*** |
| --- | --- | --- | --- | --- | --- | --- |
| • The overall organization of monitoring |  |  |  |  |  |  |
| • Your role within the study **^1^** |  |  |  |  |  |  |
| • The acceptability of the method and motivation of investigators to participate |  |  |  |  |  |  |
| • The frequency of your trips to the centres |  |  |  |  |  |  |
| • The usefulness of your trips |  |  |  |  |  |  |
| • The number of queries and mistakes |  |  |  |  |  |  |
| • The availability of the CRF for consulting when monitoring |  |  |  |  |  |  |
| • Communication and traceability of errors |  |  |  |  |  |  |
| • Other (specify): |  |  |  |  |  |  |

* Not Applicable.

**^1^**responsibility, allocation of tasks …

**b. If you are dissatisfied with the frequency of your trips, it is because:**

It is too high

It is too low

1. **a. Which eCRF creation software programs were used in your studies?**

CleanWEB

Other (specify):

**b. Which one do you prefer?**

CleanWEB

Other (specify):

No preference

**c. If you prefer a particular software, what is/are the reason(s)?**

|  | **Yes** | **No** | **NA** |
| --- | --- | --- | --- |
| • The investigators prefer it |  |  |  |
| • The interface is more ergonomic |  |  |  |
| • Immediate checks and constraints are more efficient |  |  |  |
| • A shorter timeframe for resolution technical problems |  |  |  |
| • Other (specify): |  |  |  |

**C- YOUR OPINIONS ABOUT THE PAPER CRF DATA COLLECTION METHOD**

1. **If you have experience with pCRF data collection, how would you characterize your overall satisfaction?**

Very unsatisfied  Fairly unsatisfied  No opinion  Fairly satisfied  Very satisfied

1. **a. What do you think more precisely about pCRF?**

| With respect to: | **1**  **Very unsatisfied** | **2**  **Unsatisfied** | **3**  **No opinion** | **4**  **Satisfied** | **5**  **Very satisfied** | **NA*** |
| --- | --- | --- | --- | --- | --- | --- |
| • The overall organization of monitoring |  |  |  |  |  |  |
| • Your role within the study **^1^** |  |  |  |  |  |  |
| • The acceptability of the method and motivation of investigators to participate |  |  |  |  |  |  |
| • The frequency of your trips |  |  |  |  |  |  |
| • The usefulness of your trips to the centres |  |  |  |  |  |  |
| • The number of queries and mistakes |  |  |  |  |  |  |
| • The availability of the CRF for consulting when monitoring |  |  |  |  |  |  |
| • Communication and traceability of errors |  |  |  |  |  |  |
| • Other (specify): |  |  |  |  |  |  |

* Not Applicable.

**^1^**responsibilities, allocation of tasks …

**b. If you are dissatisfied with the frequency of your trips, it is because:**

It is too high

It is too low

**D- COMPARISONS OF THE TWO DATA COLLECTION METHODS**

1. **a. If you have experience with both data collection methods, eCRF and pCRF, which one do you prefer?**

eCRF  pCRF No opinion Depends on the situation

1. **If you prefer the eCRF, what are the reasons?**

|  | **Yes** | **No** | **NA** |
| --- | --- | --- | --- |
| • Greater acceptability of the method and motivation of investigators to participate |  |  |  |
| • The variety of computer media (digital notepad, digital-pen, notebook…) |  |  |  |
| • Time savings for the study |  |  |  |
| • Faster completion of the CRF |  |  |  |
| • Better organization of monitoring |  |  |  |
| • Increased effectiveness of your monitoring visits |  |  |  |
| • The ability to monitor data collection from the office |  |  |  |
| • CRF readily available for consulting when monitoring |  |  |  |
| • The security of immediate checks and constraints during completion |  |  |  |
| • Lower rate of queries |  |  |  |
| • Better traceability of information |  |  |  |
| • Easy extraction of target data |  |  |  |
| • Easy electronic storage (emails for inclusion…) |  |  |  |
| • Greater efficiency in logistics of resupplying treatments (email alerts …) |  |  |  |
| • Other (specify): |  |  |  |

1. **If you prefer the pCRF, what are the reasons?**

|  | **Yes** | **No** | **NA** |
| --- | --- | --- | --- |
| • Greater acceptability of the method and motivation of investigators to participate |  |  |  |
| • The concrete aspect of paper |  |  |  |
| • Time savings for the study |  |  |  |
| • Faster completion of the CRF |  |  |  |
| • Better organization of monitoring |  |  |  |
| • Greater effectiveness of your monitoring visits |  |  |  |
| • The ability to spend more time in the centres to monitor the data collection |  |  |  |
| • The lower rate of queries |  |  |  |
| • Better traceability of informations |  |  |  |
| • No technical problems |  |  |  |
| • Other (specify): |  |  |  |

1. **If your opinion depends on the situation, which method would you prefer for each kind of study:**

|  | **eCRF** | **pCRF** | **No opinion** |
| --- | --- | --- | --- |
| • Monocentric studies, few patients, few variables |  |  |  |
| • Monocentric studies, few patients, many variables |  |  |  |
| • Monocentric studies, many patients, few variables |  |  |  |
| • Monocentric studies, many patients, many variables |  |  |  |
| • Multicentric studies, few patients, few variables |  |  |  |
| • Multicentric studies, few patients, many variables |  |  |  |
| • Multicentric studies, many patients, few variables |  |  |  |
| • Multicentric studies, many patients, many variables |  |  |  |

**Based on your responses in the above table:**

**If you think the number of patients is important, what do you think is the limit?**

      patients.

**If you think the number of variables is important, what do you think is the limit?**

      variables.

**If you prefer the eCRF for multicentric studies, how many centers must there be to make it worthwhile?**

      centers.

**E- OPEN-ENDED RESPONSES**

1. **In your opinion, what would be, in order of importance, the key features of an optimal data collection method in a clinical study?**


5. **If you wish to address a topic not covered in this questionnaire, but which you think is important about these two modes of data collection in a clinical trial, please note it, explaining your reasoning:**

|  |
| --- |
