# Supplementary material for: Comparison of two data collection processes in clinical studies: electronic and paper case report forms
Source: BMC Med Res Methodol. 2014 Jan 17;14:7. doi: 10.1186/1471-2288-14-7 (PMC3909932; doi:10.1186/1471-2288-14-7)
Supplement: Additional file 5 — Analysis of investigators’ satisfaction and preferences. [file 1471-2288-14-7-S5.doc]

**Table S4. Analysis of investigators’ satisfaction and preferences (n=34).**

# PCRF global satisfaction (n=33)

| **pCRF Satisfaction** | **Experience** | | |
| --- | --- | --- | --- |
|  | **Both experiences** | **No pCRF experience** | **No eCRF experience** |
| **Missing** | 0 | 1 | 1 |
| **Not missing** | 28 | 0 | 4 |

**Studied probability: Dissatisfied versus satisfied or no opinion**

| **Variable** | **n** | **Characteristics** | **OR** | **CI (95%)** | **p** |
| --- | --- | --- | --- | --- | --- |
| **Age** | **32** | **30 to 40 years old** | 0,33 | 0,03 - 4,04 | 0,66 |
|  |  | **40 to 50 years old** | 0,93 | 0,16 - 5,54 |  |
|  |  | **> 50 years old** | 1 | - |  |
| **Gender** | **32** | **Male** | 1 | - | 0,41 |
|  |  | **Female** | 0,47 | 0,08 - 2,81 |  |
| **Clinical study using pCRF in the past 5 years** | **32** | **1 to 5** | 1,78 | 0,25 - 12,45 | 0,82 |
|  |  | **6 to 10** | 1,14 | 0,13 - 10,38 |  |
|  |  | **>10** | 1 |  |  |
| **Current computer proficiency level** | **32** | **Average** | 1,19 | 0,23 - 6,17 | 0,84 |
|  |  | **Good** | 1 | - |  |
| **Participate in a study to:** | | | | | |
| **Standardize health care protocols in your specialty** | **29** | **Not at all** | 0,50 | 0,04 - 5,74 | 0,77 |
|  |  | **A little** | 0,57 | 0,09 - 3,83 |  |
|  |  | **Very much** | 1 | - |  |
| **Improve care in your specialty** | **29** | **Not at all or a little** | 1,36 | 0,11 - 17,42 | 0,81 |
|  |  | **Very much** | 1 | - |  |
| **Enhance your work** | **29** | **Not at all** | 0,75 | 0,05 - 11,31 | 0,87 |
|  |  | **A little** | 1,36 | 0,20 - 9,28 |  |
|  |  | **Very much** | 1 | - |  |
| **Develop interprofessional relationships** | **29** | **Not at all or a little** | 0,71 | 0,10 - 4,89 | 0,72 |
|  |  | **Very much** | 1 | - |  |
| **Earn additional income** | **29** | **Not at all** | 0,50 | 0,07 - 3,74 | 0,50 |
|  |  | **A little** | 1 | - |  |
| **Develop professional opportunities** | **29** | **Not at all** | 0,30 | 0,01 - 6,38 | 0,22 |
|  |  | **A little** | 2,25 | 0,19 - 27,37 |  |
|  |  | **Very much** | 1 | - |  |

**ECRF global satisfaction (n=29)**

| **eCRF Satisfaction** | **Experience** | | |
| --- | --- | --- | --- |
|  | **Both experiences** | **No pCRF experience** | **No eCRF experience** |
| **Missing** | 0 | 0 | 5 |
| **Not missing** | 27 | 1 | 0 |

**Studied probability: Unsatisfied versus satisfied or no opinion**

| **Variable** | **n** | **Characteristics** | **OR** | **CI (95%)** | **p** |
| --- | --- | --- | --- | --- | --- |
| **Age** | **28** | **30 to 40 years old** | 4,00 | 0,27 - 58,56 | 0,60 |
|  |  | **40 to 50 years old** | 2,40 | 0,21 - 27,72 |  |
|  |  | **> 50 years old** | 1 | - |  |
| **Gender** | **28** | **Male** | 1 | - | 0,55 |
|  |  | **Female** | 1,75 | 0,28 - 10,81 |  |
| **Clinical study using eCRF in the past 5 years** | **28** | **1 to 5** | 0,90 | 0,07 - 12,18 | 0,94 |
|  |  | **6 to 10** | 0,67 | 0,04 - 10,25 |  |
|  |  | **>10** | 1 | - |  |
| **Current computer proficiency level** | **28** | **Average** | 0,29 | 0,04 - 1,92 | 0,20 |
|  |  | **Good** | 1 | - |  |
| **Participate in a study to:** | | | | | |
| **Standardize health care protocols in your specialty** | **26** | **Not at all** | 4,33 | 0,21 - 90,85 | 0,27 |
|  |  | **A little** | 7,80 | 0,65 - 93,81 |  |
|  |  | **Very much** | 1 | - |  |
| **Improve care in your specialty** | **Not calculable** | | | | |
| **Enhance your work** | **26** | **Not at all** | 1,50 | 0,12 - 18,44 | 0,75 |
|  |  | **A little or very much** | 1 | - |  |
| **Develop interprofessional relationships** | **26** | **Not at all** | 2,83 | 0,35 - 23,01 | 0,33 |
|  |  | **A little or very much** | 1 | - |  |
| **Earn additional income** | **Not calculable** | | | | |
| **Develop professional opportunities** | **26** | **Not at all** | 4,80 | 0,62 - 37,35 | 0,13 |
|  |  | **A little or very much** | 1 | - |  |

# Global preference (n=28)

| **CRF preference** | **Experience** | | |
| --- | --- | --- | --- |
|  | **Both experience** | **No pCRF experience** | **No eCRF experience** |
| **Missing** | 1 | 1 | 5 |
| **Not missing** | 27 | 0 | 0 |

| **Variable** | **n** | **Studied probability** | **Characteristics** | **OR** | **CI (95%)** | **p** |
| --- | --- | --- | --- | --- | --- | --- |
| **Age** | **27** | **No opinion vs. eCRF** | **30 to 40 years old** | 1,67 | 0,15 - 18,87 | 0,83 |
|  |  |  | **40 to 50 years old** | 1,67 | 0,25 - 11,07 |  |
|  |  |  | **> 50 years old** | 1 | - |  |
|  |  | **pCRF vs. eCRF** | **30 to 40 years old** | 5,00 | 0,27 - 91,52 |  |
|  |  |  | **40 to 50 years old** | 2,00 | 0,13 - 29,81 |  |
|  |  |  | **> 50 years old** | 1 | - |  |
| **Gender** | **27** | **No opinion vs. eCRF** | **Male** | 1 | - | 0,54 |
|  |  |  | **Female** | 0,35 | 0,05 - 2,41 |  |
|  |  | **pCRF vs. eCRF** | **Male** | 1 | - |  |
|  |  |  | **Female** | 0,93 | 0,11 - 7,82 |  |
| **Clinical study using pCRF in the past 5 years** | **27** | **No opinion vs. eCRF** | **1 to 5** | 0,60 | 0,04 - 8,73 | 0,68 |
|  |  |  | **6 to 10** | 0,17 | 0.01 - 2.98 |  |
|  |  |  | **>10** | 1 | - |  |
|  |  | **pCRF vs. eCRF** | **1 to 5** | 0,40 | 0,02 - 10,02 |  |
|  |  |  | **6 to 10** | 0,33 | 0,01 - 8,18 |  |
|  |  |  | **>10** | 1 | - |  |
| **Clinical study using eCRF in the past 5 years** | **27** | **No opinion vs. eCRF** | **1 to 5** | 0,10 | 0,01 - 1,29 | 0,30 |
|  |  |  | **6 to 10** | 0,08 | 0,01 - 1,19 |  |
|  |  |  | **>10** | 1 | - |  |
|  |  | **pCRF vs. eCRF** | **1 to 5** | 0,06 | 0,00 - 1,23 |  |
|  |  |  | **6 to 10** | 0,07 | 0,00 - 1,51 |  |
|  |  |  | **>10** | 1 | - |  |
| **Current computer proficiency level** | **27** | **No opinion vs. eCRF** | **Average** | 0,20 | 0,03 - 1,42 | 0,16 |
|  |  |  | **Good** | 1 | - |  |
|  |  | **pCRF vs. eCRF** | **Average** | 0,13 | 0,01 - 1,39 |  |
|  |  |  | **Good** | 1 | - |  |

| **Variable** | **n** | **Studied probability** | **Characteristics** | **OR** | **CI (95%)** | **p** |
| --- | --- | --- | --- | --- | --- | --- |
| **Participate in a study to:** | | | | | | |
| **Standardize health care protocols in your specialty** | **26** | **No opinion vs. eCRF** | **Not at all** | 9,00 | 0,66 - 122,79 | 0,13 |
|  |  |  | **A little** | 12,00 | 0,94 - 153,78 |  |
|  |  |  | **Very much** | 1 | - |  |
|  |  | **pCRF vs. eCRF** | **Not at all** | 9,00 | 0,28 - 285,48 |  |
|  |  |  | **A little** | 26,99 | 1,26 - 577,94 |  |
|  |  |  | **Very much** | 1 | - |  |
| **Improve care in your specialty** | **Not calculable** | | | | | |
| **Enhance your work** | **26** | **No opinion vs. eCRF** | **Not at all** | 2,50 | 0,19 - 32,80 | 0,39 |
|  |  |  | **A little or very much** | 1 | - |  |
|  |  | **pCRF vs. eCRF** | **Not at all** | 6,67 | 0,44 - 101,72 |  |
|  |  |  | **A little or very much** | 1 | - |  |
| **Develop interprofessional relationships** | **26** | **No opinion vs. eCRF** | **Not at all** | 6,66 | 0,60 - 74,46 | 0,27 |
|  |  |  | **A little or very much** | 1 | - |  |
|  |  | **pCRF vs. eCRF** | **Not at all** | 6,66 | 0,44 - 101,67 |  |
|  |  |  | **A little or very much** | 1 | - |  |
| **Earn additional income** | **Not calculable** | | | | | |
| **Develop professional opportunities** | **26** | **No opinion vs. eCRF** | **Not at all** | 3,00 | 0,41 - 21,88 | 0,27 |
|  |  |  | **A little or very much** | 1 | - |  |
|  |  | **pCRF vs. eCRF** | **Not at all** | 6,75 | 0,64 - 71,17 |  |
|  |  |  | **A little or very much** | 1 | - |  |
