# Supplementary material for: Comparison of two data collection processes in clinical studies: electronic and paper case report forms
Source: BMC Med Res Methodol. 2014 Jan 17;14:7. doi: 10.1186/1471-2288-14-7 (PMC3909932; doi:10.1186/1471-2288-14-7)
Supplement: Additional file 6 — Analysis of CRAs and DMs’ satisfaction and preferences. [file 1471-2288-14-7-S6.doc]

# Table S5. Analysis of CRAs and DMs’ satisfaction and preferences (n=59).

# PCRF global satisfaction (n=57)

| **pCRF Satisfaction** | **Experience** | | |
| --- | --- | --- | --- |
|  | **Both experiences** | **No pCRF experience** | **No eCRF experience** |
| **Missing** | 5 | 2 | 0 |
| **Not missing** | 39 | 9 | 13 |

**Studied probability: Dissatisfied versus satisfied or no opinion**

| **Variable** | **n** | **Characteristics** | **OR** | **CI (95%)** | **p** |
| --- | --- | --- | --- | --- | --- |
| **Age** | **51** | **< 30 years old** | 0,67 | 0,05 - 8,73 | 0,29 |
|  |  | **30 to 40 years old** | 2,47 | 0,25 - 24,46 |  |
|  |  | **> 40 years old** | 1 | - |  |
| **Gender** | **52** | **Male** | 1 | - | 0,21 |
|  |  | **Female** | 4,03 | 0,46 - 35,23 |  |
| **Experience in clinical research** | **52** | **< 1 year** | 1,67 | 0,11 - 25,44 | 0,96 |
|  |  | **1 to 3 years** | 1,05 | 0,16 - 6,78 |  |
|  |  | **3 to 5 years** | 1,50 | 0,21 - 11,00 |  |
|  |  | **> 5 years** | 1 | - |  |
| **Clinical study participation in the past 5 years** | **52** | **1 to 5** | 1,71 | 0,29 - 10,20 | 0,78 |
|  |  | **6 to 10** | 1,95 | 0,27 - 13,98 |  |
|  |  | **>10** | 1 | - |  |
| **Profession** | **52** | **ARC** | 1 | - | 0,59 |
|  |  | **DM** | 0,63 | 0,12 - 3,38 |  |

# ECRF global satisfaction (n=46)

| **eCRF Satisfaction** | **Experience** | | |
| --- | --- | --- | --- |
|  | **Both experiences** | **No pCRF experience** | **No eCRF experience** |
| **Missing** | 1 | 0 | 13 |
| **Not missing** | 43 | 2 | 0 |

**Studied probability: Unsatisfied versus satisfied or no opinion**

| **Variable** | **n** | **Characteristics** | **OR** | **CI (95%)** | **p** |
| --- | --- | --- | --- | --- | --- |
| **Age** | **44** | **< 30 years old** | 0,25 | 0,03 - 2,36 | 0,24 |
|  |  | **30 to 40 years old** | 1,08 | 0,16 - 7,42 |  |
|  |  | **> 40 years old** | 1 | - |  |
| **Gender** | **45** | **Male** | 1 | - | 0,73 |
|  |  | **Female** | 1,30 | 0,29 - 5,86 |  |
| **Experience in clinical research** | **45** | **< 1 year** | 1,78 | 0,19 - 16,49 | 0,88 |
|  |  | **1 to 3 years** | 0,95 | 0,18 - 5,08 |  |
|  |  | **3 to 5 years** | 0,67 | 0,09 - 5,13 |  |
|  |  | **> 5 years** | 1 | - |  |
| **Clinical study participation in the past 5 years** | **44** | **1 to 5** | 1,09 | 0,09 - 13,77 | 0,72 |
|  |  | **6 to 10** | 1,88 | 0,18 - 19,66 |  |
|  |  | **>10** | 1 | - |  |
| **Profession** | **45** | **ARC** | 1 | - | 1,00 |
|  |  | **DM** | 1,00 | 0,25 - 4,06 |  |

# Global preference (n=44)

| **eCRF Satisfaction** | **Experience** | | |
| --- | --- | --- | --- |
|  | **Both experiences** | **No pCRF experience** | **No eCRF experience** |
| **Missing** | 1 | 2 | 13 |
| **Not missing** | 43 | 0 | 0 |

| **Variable** | **n** | **Studied probability** | **Characteristics** | **OR** | **CI (95%)** | **p** |
| --- | --- | --- | --- | --- | --- | --- |
| **Age** | **42** | **No opinion vs. eCRF** | **< 30 years old** | 0,30 | 0,04 - 2,42 | 0,19 |
|  |  |  | **30 to 40 years old** | 1,07 | 0,13 - 8,79 |  |
|  |  |  | **> 40 years old** | 1 | - |  |
|  |  | **pCRF vs. eCRF** | **< 30 years old** | 0,18 | 0,01 - 4,26 |  |
|  |  |  | **30 to 40 years old** | 2,40 | 0,17 - 34,93 |  |
|  |  |  | **> 40 years old** | 1 | - |  |
| **Gender** | **43** | **No opinion vs. eCRF** | **Male** | 0,60 | 0,11 - 3,21 | 0,12 |
|  |  |  | **Female** | 1 | - |  |
|  |  | **pCRF vs. eCRF** | **Male** | 0,16 | 0,03 - 0,98 |  |
|  |  |  | **Female** | 1 | - |  |
| **Experience in clinical research** | **43** | **No opinion vs. eCRF** | **< 3 years** | 0,25 | 0,05 - 1,37 | 0,46 |
|  |  |  | **3 to 5 years** | 0,67 | 0,09 - 5,13 |  |
|  |  |  | **> 5 years** | 1 | - |  |
|  |  | **pCRF vs. eCRF** | **< 3 years** | 0,50 | 0,06 - 4,15 |  |
|  |  |  | **3 to 5 years** | 1,50 | 0,14 - 16,54 |  |
|  |  |  | **> 5 years** | 1 | - |  |
| **How many studies using eCRF in the past 5 years** | **42** | **No opinion vs. eCRF** | **1** | 1,00 | 0,23 - 4,28 | 1,00 |
|  |  |  | **2 and more** | 1 | - |  |
|  |  | **pCRF vs. eCRF** | **1** | 1,00 | 0,18 - 5,46 |  |
|  |  |  | **2 and more** | 1 | - |  |
| **How many studies using pCRF in the past 5 years** | **42** | **No opinion vs. eCRF** | **1 to 5** | 0,11 | 0,01 - 1,17 | 0,18 |
|  |  |  | **6 to 10** | 0,06 | 0,01 - 0,76 |  |
|  |  |  | **>10** | 1 | - |  |
|  |  | **pCRF vs. eCRF** | **1 to 5** | 0,11 | 0,01 - 1,34 |  |
|  |  |  | **6 to 10** | 0,03 | 0,00 - 0,64 |  |
|  |  |  | **>10** | 1 | - |  |
| **Profession** | **43** | **No opinion vs. eCRF** | **ARC** | 1 | - | 0,56 |
|  |  |  | **DM** | 0,52 | 0,12 - 2,29 |  |
|  |  | **pCRF vs. eCRF** | **ARC** | 1 | - |  |
|  |  |  | **DM** | 1,26 | 0,25 - 6,36 |  |
